# Soft Robots Manufacturing: A Review

**DOI:** 10.3389/frobt.2018.00084

**Published:** 2018-07-31

**Authors:** François Schmitt, Olivier Piccin, Laurent Barbé, Bernard Bayle

**Affiliations:** ICube laboratory, University of Strasbourg/INSA Strasbourg/CNRS, Strasbourg, France

**Keywords:** soft robotics, manufacturing process, soft components, prototyping, design

## Abstract

The growing interest in soft robots comes from the new possibilities offered by these systems to cope with problems that cannot be addressed by robots built from rigid bodies. Many innovative solutions have been developed in recent years to design soft components and systems. They all demonstrate how soft robotics development is closely dependent on advanced manufacturing processes. This review aims at giving an insight on the current state of the art in soft robotics manufacturing. It first puts in light the elementary components that can be used to develop soft actuators, whether they use fluids, shape memory alloys, electro-active polymers or stimuli-responsive materials. Other types of elementary components, such as soft smart structures or soft-rigid hybrid systems, are then presented. The second part of this review deals with the manufacturing methods used to build complete soft structures. It includes molding, with possibly reinforcements and inclusions, additive manufacturing, thin-film manufacturing, shape deposition manufacturing, and bonding. The paper conclusions sums up the pros and cons of the presented techniques, and open to developing topics such as design methods for soft robotics and sensing technologies.

## 1. Introduction

The interest in soft robots has significantly increased in recent years. This evolution is more than just a trend. The scientific community is seeking to carry out a real technological breakthrough, justified by the need to evolve toward human friendly robotics. Industrial robots are fast and precise systems, based on rigid-body mechanisms, which ensure high throughput in the production of manufactured goods. The further development of robotic manufacturing now relies on the integration of workers in the manufacturing systems, allowing to perform tasks that require cognitive capacities still beyond the reach of artificial systems. In this context, collaborative manipulation has been a noticeable evolution in recent years. Industrial robots of a new type have appeared, with design and control strategies focused on the ability to perform safe physical human-robot interactions. In parallel, the development of flexible systems in robotics, e.g., serial elastic actuators, has also contributed to the emergence of new mechanisms, with similar safety and interaction objectives. If this new generations of robots has sometimes been referred to as soft robotics (Albu-Schaffer et al., 2008), such systems are still rigid link robots, which embed sensing and control capabilities allowing to operate more safely in a human environments. Soft robots are a step further in the attempt to benefit from mechanical compliance in order to offer safety and, simultaneously, approach the incredible capabilities of evolved living systems in complex tasks.

**Figure 1:**
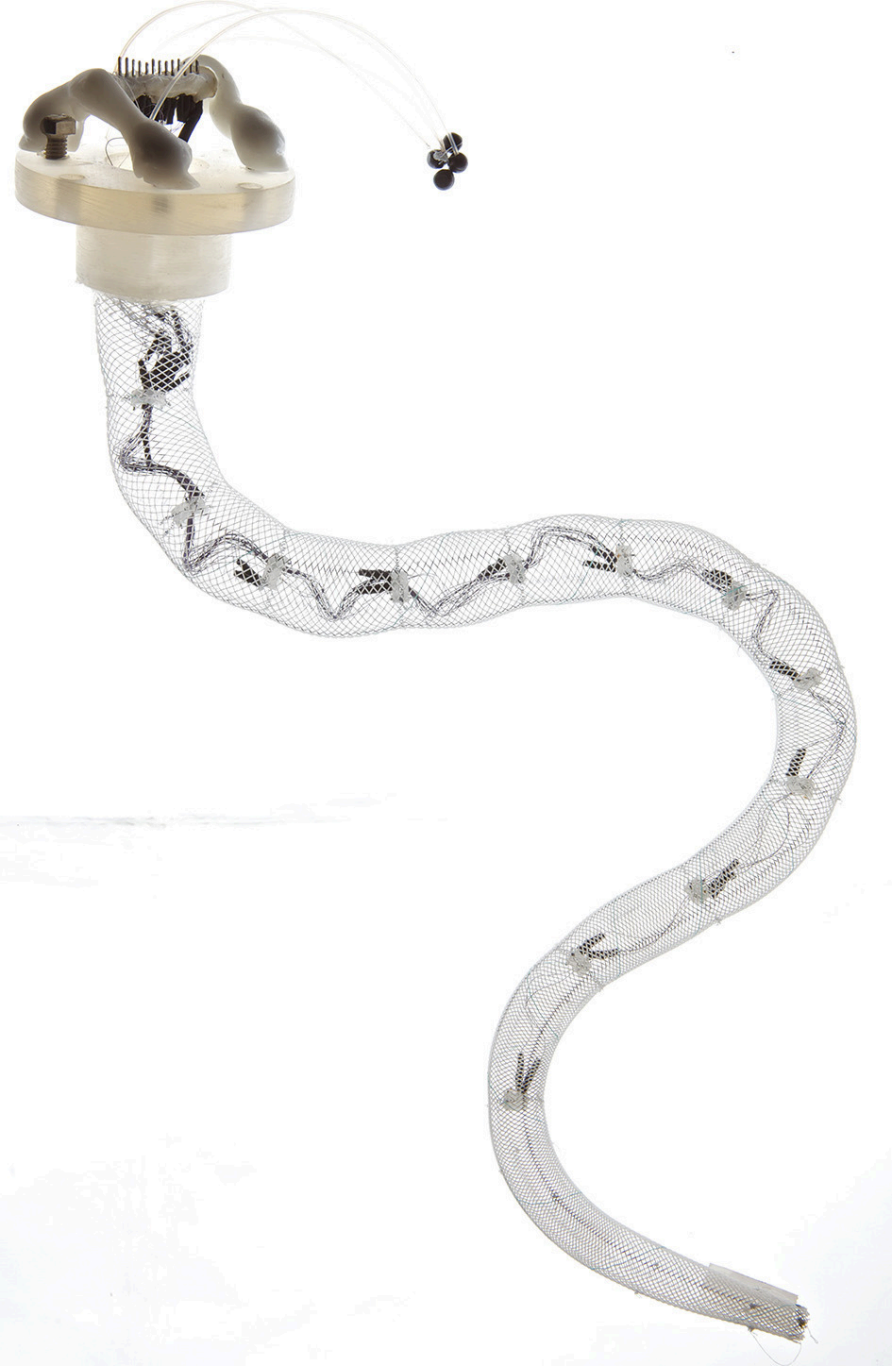
Example of a bioinspired soft octopus tentacle (Laschi et al., 2012), based on a braided polymeric network that can be constricted using SMA actuators. Image courtesy of C. Laschi and M. Cianchetti, reproduced with permission.

Bio-inspiration, which has long been controversial in the robotics community, is certainly one of the precursors of soft robotics. Bio-inspired systems, which mimic animal or human capabilities, have however long been designed using mostly rigid-body architectures, associated to soft parts. Pioneer works in soft robotics are to be found in research efforts that proposed to simultaneously inspire themselves from natural properties, and to imagine innovative ways to produce them (see Figure 1). Cho et al. (2009), in a review dating back from the early period of soft robotics, well emphasized the very close relationships between manufacturing evolutions and the design and fabrication of systems of a totally novel generation. Soft robots are systems built from materials with mechanical properties similar to those of living tissues, designed and manufactured in a very innovative way rather than artificially assembled by serial or parallel arrangements of elementary blocks, as it was the case for rigid-body robots.

The enthusiasm generated by soft robotics comes from the convergence of different scientific communities for the design of these new machines. Born at the crossroads between chemistry, plastics engineering, and mechatronics (Trivedi et al., 2008; Ilievski et al., 2011), soft robots have now spread in a great number of directions, leading computer scientists to work on design processes adapted to their non conventional structural analysis, physicists and material engineers to innovate in sensing, power supply and information processing. The endless scientific and technological opportunities raised by the development of soft robotic systems has been such that a new scientific community has gathered in a very short time, leading to the apparition of SoRo in 2014, the first scientific journal dedicated to soft robotics (SORO, 2014). Since then, the recent open-access Frontiers in Robotics and AI has also opened a section dedicated to soft robotics (Frontiers, 2016), that started publishing articles in late 2016. During this same period, soft robotics has even found a greater visibility in the prestigious Nature publication, a multidisciplinary scientific journal in which robotic science and technologies are very rarely published. In this very well documented review, Rus and Tolley show the extent of research fields concerned by soft robotics developments (Rus and Tolley, 2015). The recent scientific development of soft robotics has taken place in parallel with successful popular initiatives, like the one carried out by the Soft Robotics Toolkit project (Holland et al., 2017). This project, initiated at Harvard University in order to inspire and develop skills in undergraduate design students, has expanded both geographically and in terms of audience, including contributions ranging from high school projects to research competitions.

Though soft robotics is a recent scientific and technological topic, a significant number of review articles have already been published (Trivedi et al., 2008; Ilievski et al., 2011; Laschi and Cianchetti, 2014; Majidi, 2014; Rus and Tolley, 2015; Laschi et al., 2016), justified by the exponentially growing interest in the scientific community, and by the number of excellent recent contributions. Some observations can be made, based on these literature reviews. Up to now, most of the works that have been published in soft robotics research focus on the development of elementary functionalities, underlying the development of soft robotics. Very few research has already been concerned with the development of fully functional systems, such as those mentioned in futuristic forecasts (Majidi, 2014), highlighting the fact that soft robotics is still in its infancy. In our view, it has to be particularly emphasized that soft robotics development will be very closely related to the development of advanced manufacturing processes within the robotics community, and to the overall development of new materials and manufacturing technologies. If new solutions will come from innovations in manufacturing, conversely, soft robotics development is an opportunity to boost innovation in manufacturing and design. Very few review papers focus on soft robotics manufacturing methods. Cho et al. (2009) published a review article on the subject very early in the development of soft robotics. In the present article, we propose an update on the technologies that have been used or developed during the last decade of expansion of soft robotics. It will complement other recent review papers (Rus and Tolley, 2015; Laschi et al., 2016) that cover a broader spectrum. The paper focus is deliberately limited to the manufacturing of components and systems that constitute the structures of soft robots. In spite of the numerous developing technologies for soft sensors, embedded soft electronics or soft energy sources, we chose not to include them in the article body, as their presentation would deserve much more than a section in a review paper dedicated to the manufacturing of soft robots in general.

The present State of the Art is organized as follows. In section 2, we review the elementary components that can be used to develop soft robots. They all include original properties that differentiate them totally from the building blocks of conventional robots. They offer solutions to both the design and fabrication of hybrid rigid/soft systems, or to the development of fully soft systems. Section 3 deals with the different manufacturing methods used to obtain complete soft structures, including molding, with possibly reinforcements and inclusions, additive manufacturing, shape deposition manufacturing, and bonding. The conclusion will sum up the lessons learnt from this literature review and will open to various concerns that are closely related to the problem of manufacturing soft robots. In particular it puts in light the recent research in this field, i.e., the interconnection between soft robots manufacturing and design. It also provides the reader with somes references to start the exploration of the field of soft sensors.

## 2. Soft components for robot building

### 2.1. Soft fluidic actuation

**Figure 2:**
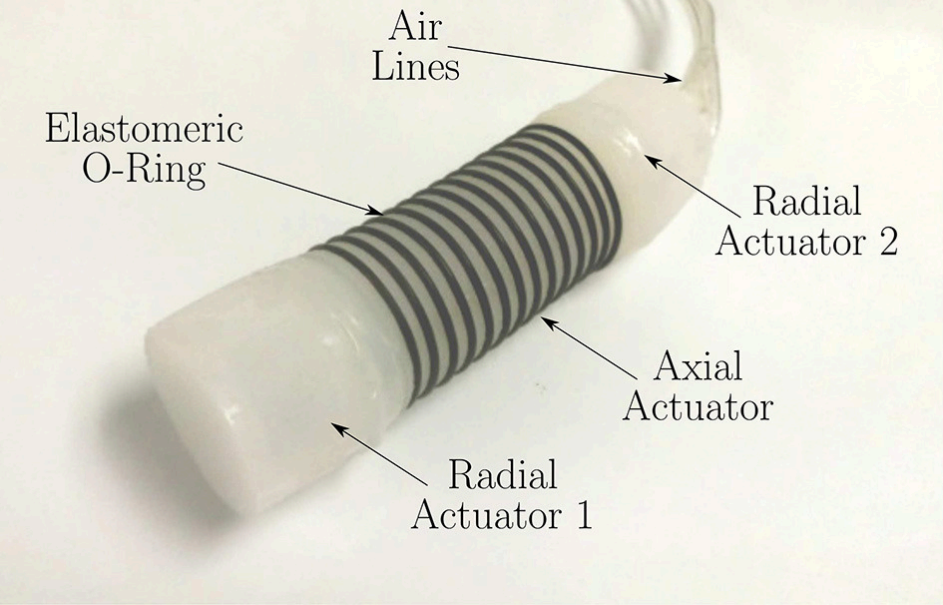
Soft worm robot as proposed in Calderón et al. (2016). The radial expansion of the axial actuator is limited by the use of stiffer o-rings. The radial actuators have not been reinforced, allowing them to expand in all the directions. Image courtesy of A. A. Calderón and N. O. Pérez-Arancibia, reproduced with permission.

**Figure 3:**
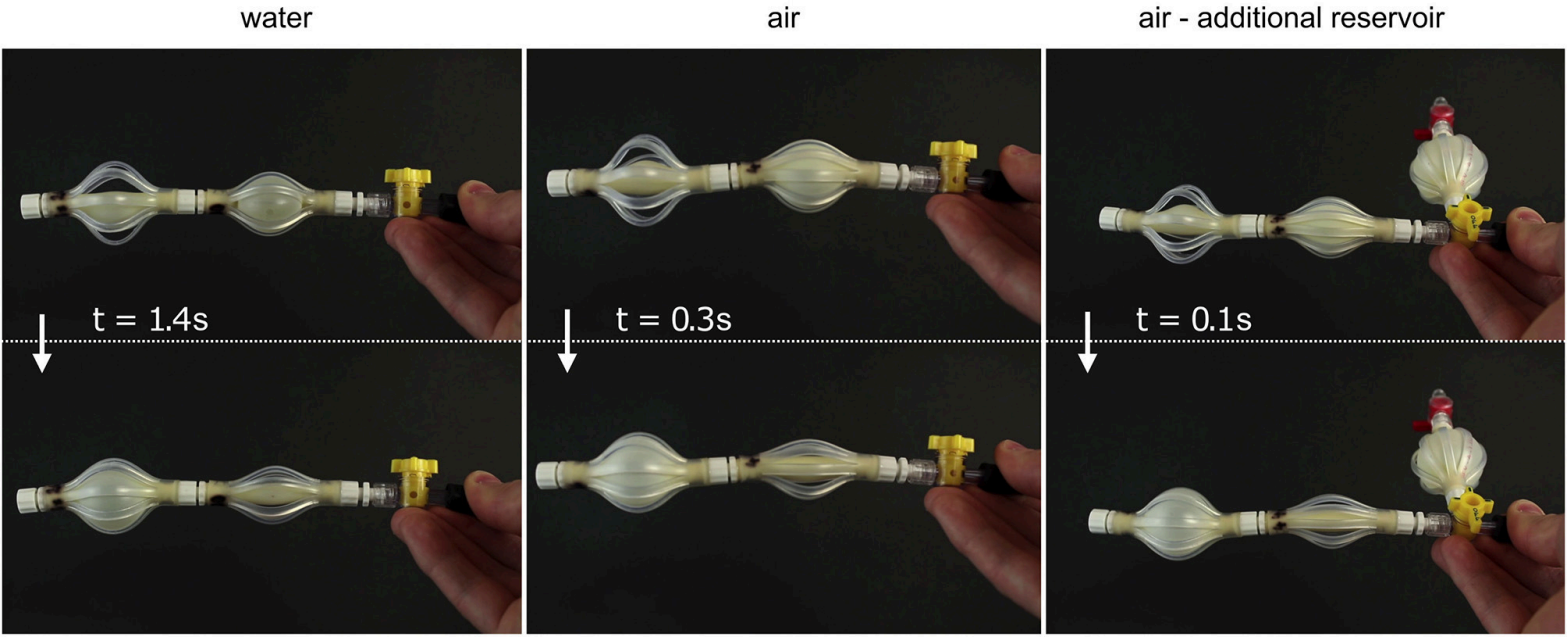
Demonstration of the fast acting system based on elastic instabilities, as presented in Overvelde et al. (2015). The soft actuator comprises two interconnected fluidic segments, with different tube and braid lengths. The actuator is first inflated with an initial volume of 16 mL, then decoupled from the syringe pump and connected to a small reservoir containing only 1 mL of water. When the system is inflated with water, it takes more than 1 s for the changes in length, pressure, and internal volume to fully take place. By replacing water with air, the time is reduced from 1.4 s to 300 ms. Moreover, by adding an additional reservoir of air to increase the energy stored in the system, the actuation time can be further decreased to 100 ms. Image courtesy of K. Bertoldi, reproduced with permission.

The easiest way to create a homogeneous load on a deformable piece of material is to raise the applied pressure by the use of some fluidic medium. This concept is at the core of a large number of soft robotic actuators and their various implementations. Simplest soft fluidic actuator designs involve bladders that may quickly be inflated with compressed air, thus generating the impulse needed for jumping robots (Ni et al., 2015), or creating fluid motion from mechanical action (Giorgio-Serchi et al., 2016) or from internal combustion (Loepfe et al., 2014; Schumacher et al., 2014). By giving the bladder a specific spatial architecture such as bellows (Tolley et al., 2014b; Digumarti et al., 2017), or by thickening its extremities (Qi et al., 2015), one expansion direction may be favored with respect to the others. Alternatively, thin anisotropic films can also be used to build bladders (Niiyama et al., 2014). Their high transverse flexibility with respect to their in plane stiffness can be used to favor deformation in specific directions. Additionally, pleats can be added to the design (Nishioka et al., 2017; Sareen et al., 2017), allowing the motion to follow prescribed trajectories. This “mechanical programming” can also be performed using materials with different stiffness values. Indeed, by limiting the radial expansion of tubular bladder using stiffer materials (Calderón et al., 2016; Robertson et al., 2016) (see Figure 2), it is possible to build actuators able to generate large linear extension. Some work has also been done to use the elastic instabilities between pre-stressed elastic tubes and compressed air to generate large displacements using small fluid volume variations (Overvelde et al., 2015) (see Figure 3).

**Figure 4:**
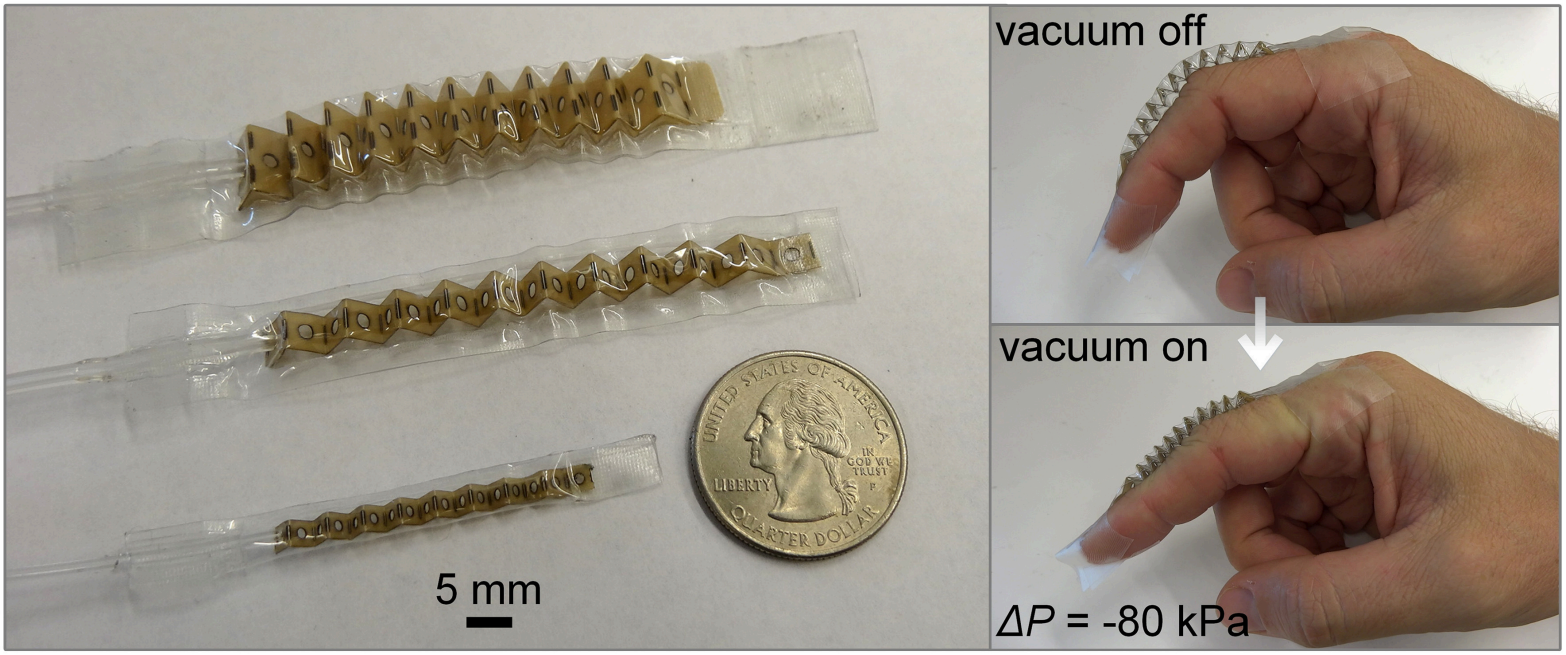
Illustrations of the origami-inspired artificial muscle proposed in Li et al. (2017a). The leftmost figure shows several actuator scales with respect to a quarter. The figures on the right presents the muscle used to pull on a finger before and after actuation using air vacuum. Image courtesy of S. Li, reproduced with permission.

Using pressurized fluid, it is also possible to obtain contraction instead of expansion. Those works have been mainly motivated by the idea of mimicking the behavior of biological muscles that contract when stimulated. In the 1950s, pneumatic artificial muscles (PAM) such as McKibben muscles (Chou and Hannaford, 1996; Tsagarakis and Caldwell, 2000), which contract when inflated with pressurized fluids, have been proposed. Since then, multiple systems and variants inspired by the McKibben PAM have been proposed, including the Pleated PAM (Daerden, 1999) or tendon driving devices (Tsujiuchi et al., 2006). Some other approaches are based on the use of vacuum in order to collapse a buckling structure (Yang et al., 2016a) or to contract a compressible skeleton using the mechanical tension of a flexible sheet on a more rigid structural element (Li et al., 2017a) (see Figure 4).

**Figure 5:**
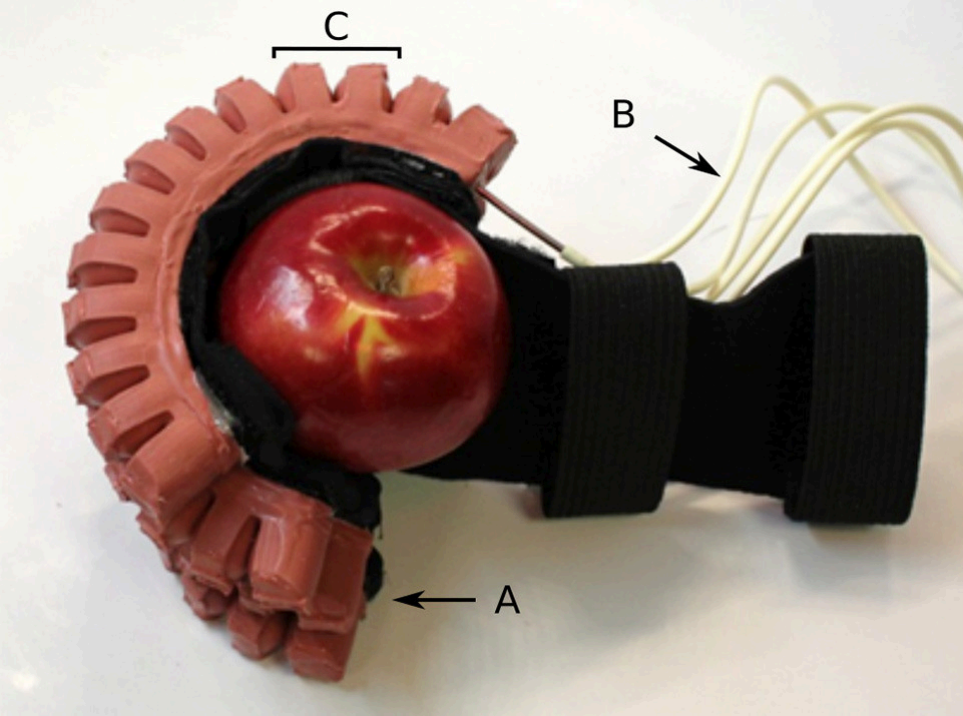
Examples of rehabilitation gloves, as proposed in Polygerinos et al. (2013). Each of the four actuated fingers **(A)** is equipped with a bellowed pneunet, which can be inflated separately using the flexible tubes **(B)**. The shape of the pneunet allows the external walls of the bellows to push on each other, as visible in **(C)**, allowing a faster actuation of the bending actuator. Image courtesy of C. J. Walsh, reproduced with permission.

Another widely studied actuator category is the family of soft fluidic bending actuators which feature a general beam-shaped geometry with an inflatable chamber along their longitudinal axis. Their bending motion results from a change of extensibility of two opposing sides of the beam. This property can also be modulated by design. The obtained actuators may exhibit a large range of motion in one or more directions depending on the number of internal chambers, their topology and the actuation method. In single-material structures, simple variations in the wall thickness leads to varying degrees of deformations under the same pressure (Gorissen et al., 2013), creating the desired bending motion. Another possibility is to reinforce one side of the actuator using a stiffer material (Polygerinos et al., 2015b). These actuators may also present bellowed geometries (Polygerinos et al., 2013; Mosadegh et al., 2014) (see Figure 5) that allow faster actuation due to a lower volume change of the internal chamber and lower strain level, which in turn increases the actuator lifetime. In order to improve the deformation along the bending direction, it is also possible to coil inextensible cables around the tubular section, thus limiting the radial deformation in favor of the axial deformation. Inflating a single chamber bending actuator usually results in a displacement in only one direction. However actuating two chambers in opposition may generate a bidirectional planar bending (Yap et al., 2016), or a multidirectional spatial bending when more chambers are available (Martinez et al., 2013). Alternatively, the planar bidirectional motion is also available when the actuator is fed either with positive or negative relative pressure (Ogura et al., 2009). It is also possible to “mechanically program” the bending motion of the actuator by varying the section of the internal chamber (Deimel and Brock, 2013, 2016), by changing the configuration of the reinforcements (Polygerinos et al., 2015a) or even by adding an external inextensible sheath (Galloway et al., 2013).

Another possible way to generate a bending motion is to create a pneumatic network (or pneunet) of small distributed chambers that can deform along the bending profile (Ilievski et al., 2011; Shepherd et al., 2011; Tolley et al., 2014a). Like tubular bending actuators, pneunets also rely on an inextensible layer to ease differential deformation. This technique has also been adapted to create actuators exhibiting specific motions such as undulatory snake-like or fish-like motion (Onal and Rus, 2012; Onal et al., 2013; Marchese et al., 2014), or even cyclic motion (Correll et al., 2014). The bending motion could also be obtained using internal explosion, creating impulsion as required by jumping robots (Shepherd et al., 2013). Additionally, taking inspiration from continuum and hyper-redundant robots, vertebra vacuum-actuated bending robots can achieve spatial bending motion (Agarwal et al., 2017). Finally, the integration of radial unstretchable layers instead of longitudinal ones makes it possible to create pure rotation instead of bending motion (Song et al., 2013; Sun et al., 2013).

Though less widespread, some methods have been proposed to create motions using fluids. Taking inspiration from stepper motors, some systems implement peristaltic motion generated by the sequential actuation of chained pneumatic chambers (Chen et al., 2014; Gong et al., 2016) or soft material integrating soft actuator in order to generate bio-inspired pumping motion similar to that of the heart (Roche et al., 2014).

While soft fluidic actuators have major advantages such as a high specific power and a wide range of available motions, their major drawback lies in the need of a fluidic power source and a complex electro-mechanical system, which may result quite bulky. If some research focused on mobile applications has led to interesting solutions to overcome these constraints (Marchese et al., 2014; Tolley et al., 2014a), this remains an issue that deserves further research efforts.

### 2.2. Alternative soft actuation methods

Material science community has developed several solutions that rely on alternative energy sources and/or activation stimuli, giving more options to soft robots designers.

Some of these smart materials rely on thermal energy in order to change their state. This is the case with materials such as Shape Memory Alloys (SMA), for which transitions in the material crystalline structure under temperature changes allow the release of stored elastic energy. Their high specific power allow them to be used in applications where compactness is critical, such as live hinges on foldable systems (Firouzeh et al., 2013), or stiffness tuning layers for soft fluidic actuators (Firouzeh et al., 2015). SMA can also act as tendon-like actuators, embedded in a rigid structure (Meisel et al., 2015) or acting as constrictive muscles for biomimetic soft tentacles (Mazzolai et al., 2012; Cianchetti et al., 2015). Similar material behavior can also be found in Shape Memory Polymers (SMP) (Yang et al., 2016b; Paulino et al., 2017). They exhibit both lower density and stiffness, limiting the energy they can release during their transition. Similarly, by using pre-stressed coiled fibrous materials, such as low cost nylon fishing line, it is possible to create thermo-activated synthetic muscle fascicles (Haines et al., 2014). Alternatively, it is also possible to use thermodynamic effects, such as phase transition, to power thermo-active actuators. From this concept, a solution using an ethanol and silicon emulsion has been proposed (Miriyev et al., 2017). This emulsion can then be shaped and used similarly to classical soft fluidic actuators, without the need of a fluid compression/distribution system, as the variation of the internal volume is obtained by the vaporization under heat of the ethanol micro-bubbles trapped in the silicon matrix.

The heat needed for these thermo-active actuator to work is generally brought by electro-resistive elements such as the material itself, for instance in the case of SMA, or the addition of resistive wiring in the structure of the actuator when its constitutive material electric conductivity is low. In order to reduce the integration complexity of soft systems, Electro-Active Polymers (EAP) have been proposed (Carrico et al., 2017a). Several families of EAP exist, such as ionic metal polymer composite (Carrico et al., 2017b), dielectric polymers/elastomers (Suo, 2010) or ionic hydrogels (Ionov, 2014). Their working principle, generally based on the migration of ionic elements under an electrical field, limits their application to thin film or small/micro scale systems as the generated forces are usually low and the response delay increases dramatically at larger scales. They allow to implement systems even at micro scales, ranging from micro-manipulators (Jager et al., 2000) to aquatic micro-walker/swimmers (Kwon Gu Han et al., 2008). Although their implementation on larger system is more limited, notable applications of these technologies are for example bending actuators for miniature vertebra (Choi et al., 2005), electro-activated muscles (Kovacs et al., 2007) or tunable lenses (Maffli et al., 2015).

Robots are generally built by associating a mechanical structure, actuators, and their control system. Smart materials such as Stimuli-Responsive Materials (SRM) allow to combine both actuation and control functions, as they passively respond to external stimuli, such as temperature, light, or chemical compounds for instance. Similarly to EAP, a wide variety of photo-responsive shape-memory and shape-changing polymers are readily available (Iqbal and Samiullah, 2013). They can also be obtained by adding light absorbing particles to classical heat-sensitive materials (Breuer et al., 2017). Other examples include smart materials that react to the presence of specific fluids, such as graphene monolayer paper (Mu et al., 2015) that react to the present of water, or inverse opal polyionic microstructures (Wu et al., 2016) that react to various solvents. Even if the use of these SRM is limited to specific application, they may effectively allow to fully integrate autonomous elements when external energy sources are scarce or on micro-scale integrated systems. Application of SRM on larger systems however is limited due to their low specific power and the difficulty of stimulus diffusion to the core of thick elements.

### 2.3. Soft smart structures

Applications of soft materials are not limited to the scope of soft actuators. Indeed, they can be used to build new structures that can have a smart behavior when exposed to specific conditions. These smart properties can either be permanent, or tunable in order to adapt the structure to an evolving environment.

The simplest of these structures are inflatable structures, which differ from fluidic actuators based on chambers by the fact that they are not actively controlled. Pressurized fluid is injected into the structure in order to transform it from a deflated to an inflated state, thus changing its mechanical properties. Several examples of such systems are found in the literature such as inflatable links (Stilli et al., 2017) or robots (Best et al., 2015; Gillespie et al., 2016) for safe human interactions, high payload-to-weight ratio manipulation arms for operation in inaccessible areas (Voisembert et al., 2013) and inflatable furniture or architecture (Sareen et al., 2017) that can deploy starting from a compact initial footprint.

**Figure 6:**
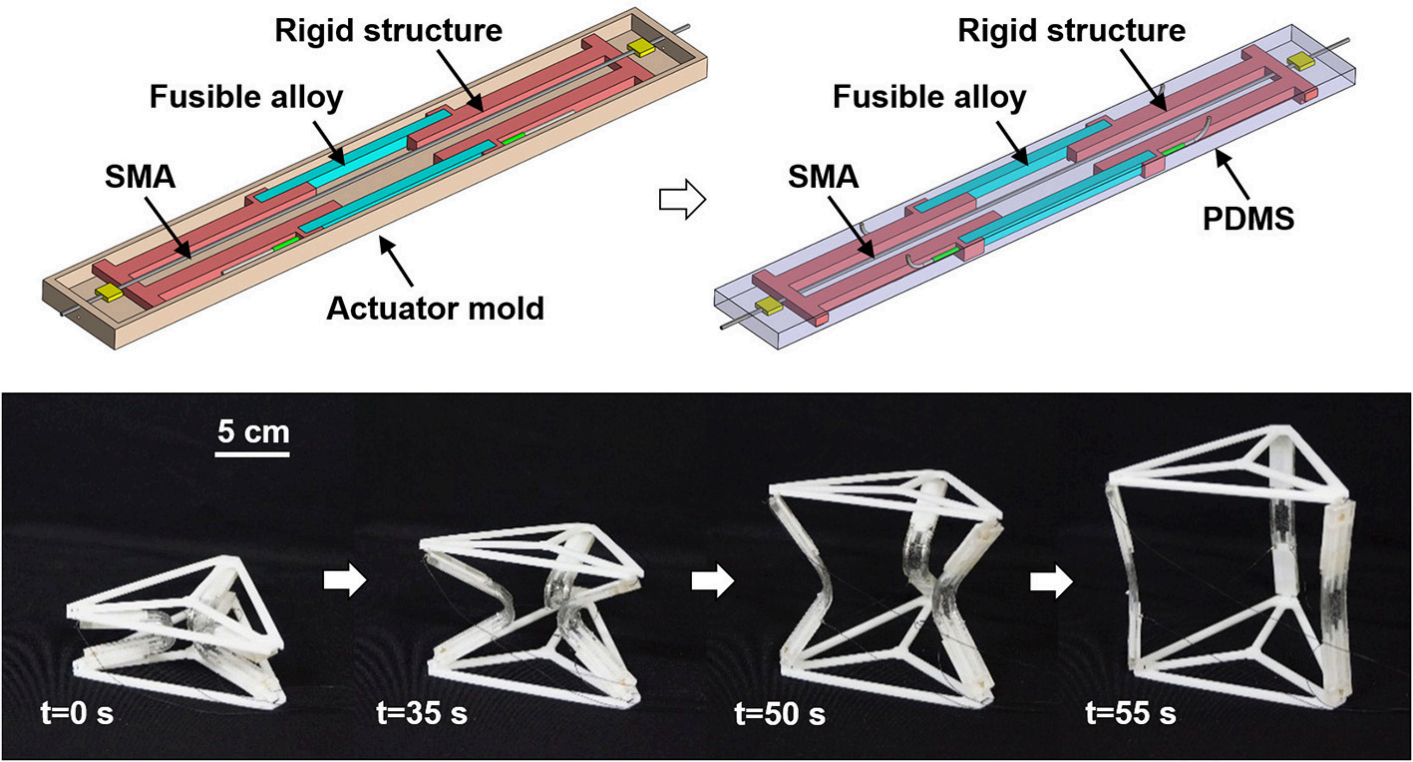
Metamorphic deployable structure (Wang et al., 2016). The top-left figure illustrates the structure assembly, before curing the polymer. The top-right figure represents the structure after curing and removing the mold. The deployment of the assembled structure is represented in the bottom figure. Image reproduced from Springer Nature with the permission of W. Wang.

Other works have been carried out to make soft structures compatible with high interaction forces occurring when manipulating or grabbing objects. From this viewpoint, the ability to switch from a soft to a stiffer structure becomes a desirable feature. In order to obtain this behavior, some systems are based on the use of low temperature fusible alloys. This allows the creation of metamorphic mobile robots that can be converted into grippers (Nakai et al., 2002), flexible deployable structures (Wang et al., 2016) (see Figure 6), tunable stiffness materials (Shan et al., 2013) or actuators (Shintake et al., 2015). Another possibility is to use particles or flat sheet layers that can be jammed together using vacuum, effectively stiffening a structure. This has been used for bending actuators (Cianchetti et al., 2014; Ranzani et al., 2016; Li et al., 2017b), continuum robots (Kim et al., 2012) or adaptive graspers (Brown et al., 2010).

Finally, auxetic structures or metamaterials are an example of purely passive structure that can be interesting from a robotic standpoint. They exhibit a negative Poisson's ratio, meaning that they expand orthogonally, instead of contracting, when uniaxially stretched. They have found some use on soft robots as passive clutch for the movement of a worm robot (Mark et al., 2016) or in the industry as shock-absorbing materials.

### 2.4. Soft-rigid hybrid systems

Until now, the majority of the presented systems were mostly based on soft materials. Their major advantages is their ability to deform when actuated and their high specific power while allowing soft interactions with their environment. However this comes also with some notable drawbacks such as low positioning accuracy, the fact they cannot generate or sustain high forces, and also the complexity of the instrumentation and control implementation. Some tasks at hand may however need the best of both soft and rigid components, combining safe interactions with high precision, or low weight with high forces, for instance. In this case, some compromises can be made, resulting in hybrid systems that are neither soft nor rigid.

While not strictly oriented toward soft robot design, some particularly interesting works have been done on stiffer materials in order to create compliant mechanisms (Wood et al., 2008; Delimont et al., 2015). Some authors also take inspiration from origami and kirigami techniques in order to design structures that exhibit both flexible motion about given axis, and a high off-axis stiffness due to mechanical overconstraints (Onal and Rus, 2013). Using specific structural patterns it is actually possible to create soft actuators exhibiting both a soft behavior and high forces (Martinez et al., 2012). Further boosted by recent developments in multimaterial additive manufacturing, these emerging concepts provide new solutions to design complex structures that can be produced with minimal amount of operations (Bruyas et al., 2015; Wang and Lee, 2017).

## 3. Shaping soft structures

Alongside the development of the technological elements presented in the previous section, in-depth work has also been done in order to propose solutions to manufacture these elements. As soft elements are mainly made of polymers, it is natural that most proposed methods have been derived from solution that have been used for plastics engineering. Consequently, proposed building methods will mainly focus on molding and adding reinforcements and inclusions of interest. Some additive manufacturing solutions will also be presented as they may offer convenient solutions in order to craft soft systems. In parallel, a consequent part of the research in soft robotics is oriented toward the design of small scale systems. As such, manufacturing methods initially developed for thin film electronics manufacturing that were adapted in order to produce soft mechanical systems will also be presented.

### 3.1. Molding

A large majority of soft structures are built using catalyzed polymer such as silicone rubbers that are obtained by mixing two component before molding operations. The homogenization steps that are needed add however air bubbles to the mix. Because they may add weaknesses to the final structure, those bubbles need to be removed, generally by vacuum degassing the mix. Alternatively, spinning the mold and using the centrifugal forces can increase the pressure gradient and degas more effectively than using only gravity (Mazzeo and Hardt, 2013). The degassing could also be forced by applying vaccuum at strategic locations of the mold during the injection of the polymer. This technique, called vacuum casting, allows to replicate details even in the sub-millimetric range (Zhao et al., 2012).

In the case of thermoplastic material molding, such as paraffin wax, high shrinkage due to thermal retraction can be observed, leading to dimensional inaccuracies or even shape warping. In order to limit these adverse effects, the material should be molded under packing pressure during the cooling phase, in order to provide additional material and compensate for the shrinkage.

Another issue appears for multi-step molding where functional interfaces, reinforcement (Polygerinos et al., 2013), actuators (Roche et al., 2014) or even electronic components (Correll et al., 2014) may be overmolded. These added components need to be correctly located and fixed to the mold, and have to adhere to the inclusion material. This operation can be facilitated by using specific primer and glues that may enclose the overmolded component and also allow adhesion with the inclusion material.

Molding is very difficult when it comes to manufacturing internal volumes and undercuts. Molds are well suited to form external shapes in semi-round or full-round, that can then be extracted by simple pulling motion. In the case of internal volumes and undercuts, however, pulling is generally not possible as the mold or the internal core would collide with the molded material. Several solutions have been developed to tackle this issue. The easiest solution is to mold the part in several subparts that can then be sealed together by gluing (Tolley et al., 2014b) or dipping (Onal and Rus, 2012) the parts in uncured material. The internal volume can also be closed in a later operation by adding a layer of uncured material (Ilievski et al., 2011; Sun et al., 2013). The seam obtained through this method may however be structurally weak because of material heterogeneity.

When more complex internal structures are needed, these method may become insufficient. In this case, an internal core is needed during molding operations, an thus the challenge becomes to extract this core after the molding operation. For tubular shapes, the solution is to simply mold the part with a core passing throughout the mold, and to plug the holes at the extremities with additional material afterwards (Martinez et al., 2013; Polygerinos et al., 2015b). Other classical shapes may take some inspiration from industrial molding: the core could be formed by multiple parts that can be disassembled and extracted by an access hole. Similarly, the core could be made of fusible or soluble material, and be destroyed after molding the part (Schumacher et al., 2014; Marchese, 2015; Lawrence et al., 2016). Another solution is to use the flexible nature of the molded part. Galloway et al. (2016) use a soft core that can be removed out of the molded part using for example vacuum to ease the process. Alternatively, instead of a fully empty volume inside the part, Argiolas et al. (2016) propose to use soft foam material crafted using soluble filler (salt) mixed with silicone. After curing, the salt is then dissolved, leaving porous internal volume. Agarwal et al. use an overmolded foam core to simplify shaping operations (Agarwal et al., 2017).

Internal volumes can also be molded from only one side. If the precision of the internal geometry is not a priority, rotational molding can be used (Zhao et al., 2015). This method involves filling and closing mold, then spinning it about two axes in order to homogeneously cover all of its surfaces with a layer of polymer. The obtained parts offer empty internal spaces without the need of any core for the molding. The main drawback of this method is that the wall thickness is not constant and tends to produce meniscus in area with large depth variations. If, on the contrary, the internal walls details are important, dip coating can be used. This methods, notably used in the industry to mold bellows, inflatable balloons and plastic gloves, works by dipping a molding core or an insert part in a bath of liquid polymer. In order to reduce risks of streaks and excessive thickness, the polymer should exhibit both proper viscosity and adhesion to its support, and should also be set shortly after dipping.

Alternatively, when one side of the part is more important than the other, infusion molding can be used (Brouwer et al., 2003). This method, developed in order to mold fiber-reinforced composites, can also be adapted to soft composites with fibrous matrix. The mold is covered on its opposite side with a plastic bag and the polymer is then pulled using vacuum through the fibrous reinforcement until completely infused. Although this method has initially been designed for molding large parts such as boat hulls, it can also be used to mold smaller parts.

### 3.2. Reinforcements

Even if the final shape of a soft robot is dependent on the intrinsic compliance of its material, and therefore not always predictable, the designer has some leverage on the behavior of the structure by modulating its stiffness along specific directions. This is generally done by adding reinforcements to the structure.

**Figure 7:**
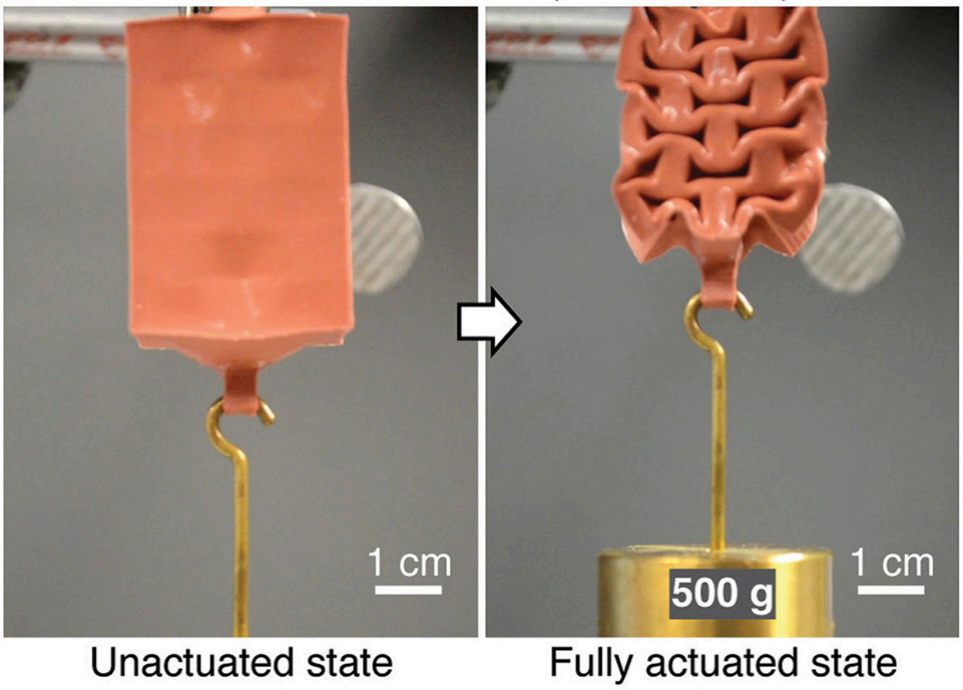
Example of a soft actuator using buckling rectangular cells to produce displacements when actuated using vacuum. Such actuators can be used as synthetic muscles on an exoskeleton as they contract similarly to biological muscles when actuated. Image reproduced from Yang et al. (2016a) with the permission of G. M. Whitesides and Wiley (Copyright Wiley-VCH Verlag GmbH & Co. KGaA).

The simplest way to reinforce a system is by modifying its geometries, modulating the overall material thickness (Gorissen et al., 2013) or adding ribs. This allows to increase stiffness in areas where deformation is not desired for the task. This is particularly important for soft fluidic actuator, as an increase in the internal pressure may lead to the extrusion of the actuator walls, and, ultimately, to the rupture of the chamber. Another possibility to avoid this pitfall is to implement bellowed geometries (Tolley et al., 2014b; Yap et al., 2016) or exploit the collapse of the material due to buckling (Yang et al., 2016a) (see Figure 7) to limit the final deformation of the shape. When modulating the geometry is not possible, the modulation of the material anisotropy may also allow to limit the deformations in specific directions. This is the case for extruded plastic films that exhibit high bending flexibility with respect to their high stiffness in traction (Niiyama et al., 2014).

**Figure 8:**
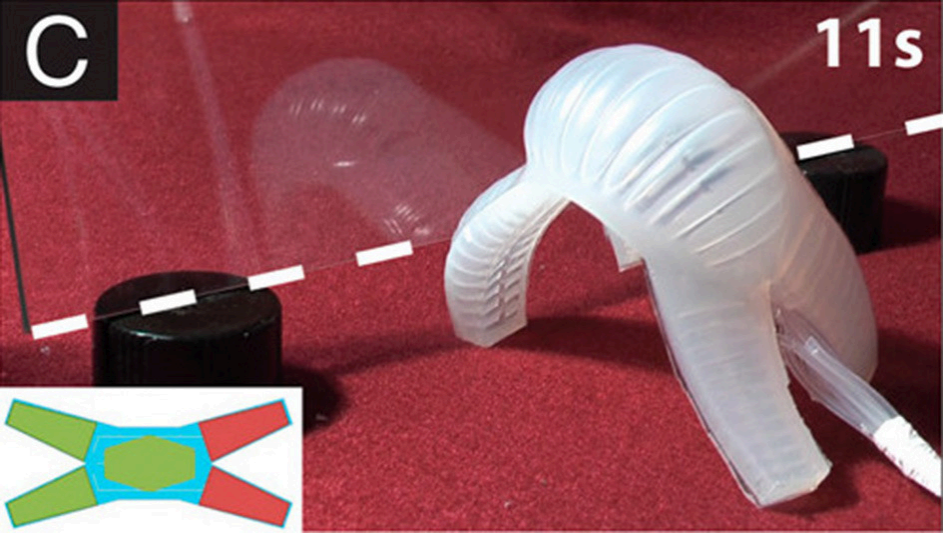
Walking robot composed of five separately inflatable pneunets molded in silicone (Shepherd et al., 2011). The strain limiting layer is composed of PDMS. Image reproduced from PNAS with the permission of G. M. Whitesides.

Modulating the flexibility of soft body parts can also be obtained by the combining materials with different mechanical properties. The combination of two (or more) heterogeneous material provides the anisotropy needed to obtain the soft mobility required while limiting transverse deformation. It is easily possible to combine materials with similar chemical composition but contrasting mechanical properties. For example, Shepherd et al. (2011) propose to use of a soft Siloxane rubber, with a shore hardness of 00-30 and an elongation at break of about 900%, as the body of an actuator, in combination with a slightly harder reinforcement, made of a PDMS rubber with a shore A hardness of 50 and an elongation at break of about 200% (see Figure 8). The contrast between stiffnesses allowed the creation of a pneumatically actuated bending grasper. Another approach is to embed in the structure a layer of highly anisotropic material such as paper (Mosadegh et al., 2014) or fabric (Sun et al., 2013) sheets. These layers exhibit very high stretching stiffness but can bend very easily, making them suitable reinforcement for bending actuators. When in opposite, the linear extension of the structure is desired, unstretchable threads can be coiled around the structure, allowing it to extend with very low diametrical expansion (Robertson et al., 2016). A combination of both a strain limiting sheet and a coiled thread can also lead to interesting designs combining twisting with bending, or even modulating the bending radius of the actuator (Polygerinos et al., 2015a).

**Figure 9:**
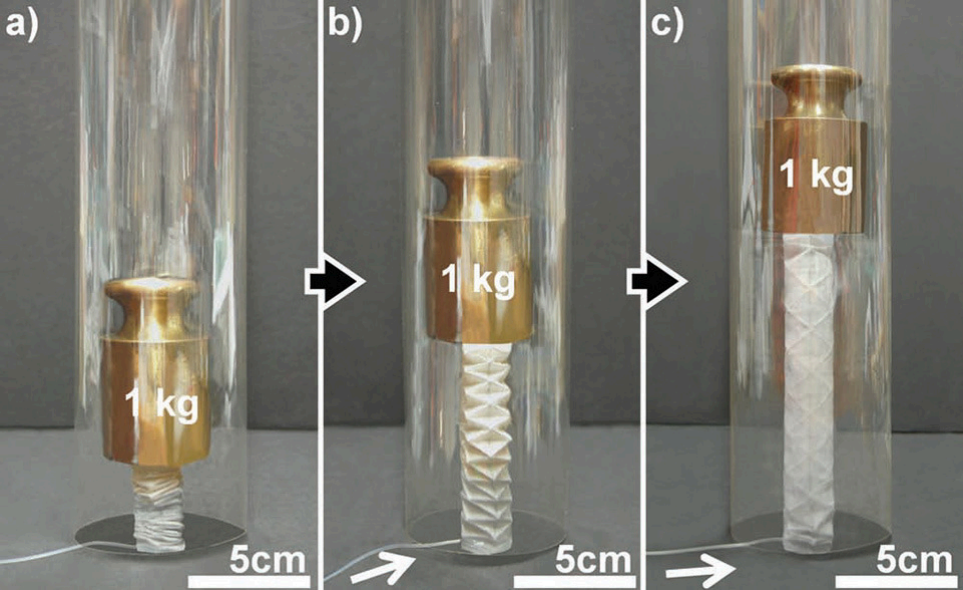
Soft linear actuator based on a silicon cylinder overmolded on a sheet of paper folded with an origami pattern. The obtained system can be actuated using pressurized air, exhibiting both large displacements and high force level. Panels **A–C** show consecutive states of the actuator during inflation. Image reproduced from Martinez et al. (2012) with the permission of G. M. Whitesides and Wiley (Copyright Wiley-VCH Verlag GmbH & Co. KGaA).

Reinforcements can be added at any time during the molding process. However, in order to avoid friction or hysteresis, they should be correctly bounded to the soft structure. Strain limiting layers could be pre-dipped in uncured polymer, and then sticked to the rest of the structure (Sun et al., 2013). The reinforcements can also be molded in the initial molding operations (Mosadegh et al., 2014) or even overmolded (Polygerinos et al., 2015b) or glued (Memarian et al., 2015b) later in the process. The silicon can also be reinforced using fillers such as short fiber or beads (Tolley et al., 2014a), modifying both the hardness and the mechanical resistance of the material. Complex behavior can also be obtained using an internal skeleton (Martinez et al., 2012) (see Figure 9), or additional external reinforcements, such as a fabric sheath (Galloway et al., 2013) or a plastic exoskeleton (Kim and Gillespie, 2015; Tao et al., 2015; Paez et al., 2016). These hybrid designs benefit both from the low stiffness of the soft core for soft interactions, and from their more rigid sections for higher forces generation. The addition of those more rigid components may also be used to enforce a motion compatible with more classical kinematics, allowing an easier transposition of dynamic models from stiff to soft robots.

### 3.3. Additive manufacturing

Unlike classical machining methods, also called subtractive manufacturing, where a tool removes scraps from a workpiece to produce the required shapes, additive manufacturing (AM) is based on the local deposition of small volumes of material in order to directly form the shape. While early AM methods were limited to the production of rigid thermoset polymers, the available technologies have been improving constantly since the early 1990's (Kruth, 1991; Kruth et al., 1998, 2005). It is now possible to create parts from polymers and possibly elastomers, metals, ceramics, and even in some cases to combine or mix two or more construction materials in a same monolithic part. This allows for instance to include electro-conductive materials in a mechanical structure in order to produce parts with potentially complex shapes that include 2D or 3D electrical circuits (Macdonald et al., 2014; Goh et al., 2017). Depending on the technology, it is possible to build parts with sizes ranging from tens of micrometers to several meters long.

Methods most commonly used for additive manufacturing are based on the selective solidification of a liquid or powdered material in order to create the desired shape. Stereolithography and selective laser sintering technologies use a laser scanning the shape of each additional layer of the desired 3D shape. In the case of stereolithography, the laser is focused in order to polymerize a small volume of pre-polymer available as a liquid bath. Selective laser sintering uses the laser to locally fuse powdered material. When each layer is finished, the construction tray is lowered and a new layer can be constructed on top of it. In the case of selective laser sintering, an additional layer of powder is added using a roller. Newer methods such as digital projection lithography (Sun et al., 2005) and continuous liquid interface production (Tumbleston et al., 2015) allow to print each layer directly, without the need of 2D-scanning, effectively speeding up the building process. Another method commonly used for AM is the fused deposition modeling (FDM) process. In this case, the 3D shape is formed by sweeping each layer using a thermoplastic extruding nozzle that creates a thin polymer string. Although parts obtained through this process usually exhibit worse spatial resolutions than those obtained through photo-activated processes, the low price of FDM machines as well as their high modularity allowed a wide adoption in the recent years by both research laboratories and competent do-it-yourselfers. Another readily available method is the inkjet printing of polymers. In this process, the printing machine is equipped with a printhead that can travel in 2D. Each material layer is printed in a fashion similar to paper desktop printers. After each layer, the printing tray is displaced as to allow the next layer to be printed. This process is a good compromise between printing speed and resolution, while keeping the production costs relatively low. Finally, halfway between FDM and inkjets, direct ink writing (Lewis, 2006) is a method that uses solidifying viscous inks to create complex shapes. Although technically more complex than the previous methods, it makes it possible to produce very small patterns, with a high control over the material orientation at each point. Unlike more classical methods, the printing direction is not restricted to one plane at a time, which permits to build shapes with anisotropic materials, such as short-fiber-filled polymers.

Comparatively to molding or machining methods, AM offers more freedom to design complex geometries. For instance, it is possible to build entanglements of convoluted shapes without any complementary part or internal core that would need to be machined using a 5-axis milling machine, if even machinable. These processes have however some limitations. Indeed, AM often relies on scaffolding in order to build precisely overhang geometries. These scaffolds can be constructed either in model material, later removed by machining, or in support material removed by mechanical (brittle support) or chemical (soluble support) action. Some manufacturing methods could be used to build overhangs without support, but in this case it is possible by either limiting the draft angle (Anver et al., 2017) or the distance between both ends of the overhang (Yap et al., 2016). Should these constraints not be respected, the obtained geometries may show excessive sagging, holes and/or mechanical weaknesses. Additionally, the scaffolding could be ignored or removed during the manufacturing process in order to include elements of different nature (Meisel et al., 2015), and then be printed over.

Additive manufacturing can also give the possibility to produce components including multiple materials. Indeed, FDM and inkjet processes may allow this multimaterial additive manufacturing by including multiple extrusion/printing heads, each loaded with different materials. Using this principle, it is possible to create mechanical device combining soft deformable parts with more rigid elements, or even to pattern both materials in order to obtain intermediary compounds (Bartlett et al., 2015). These methods may open new pathways for hybrid soft-rigid robotic systems, benefiting both from soft compliant elements and structural reinforcements, similarly to what can be found in the nature. The contrast in material properties is also not limited to mechanical properties. Finally, new manufacturing methods based on AM methods but using smart materials such as shape memory polymers also emerge. These so-called 4D-printed or shape-morphing systems are mechanical systems obtained through classical multimaterial additive manufacturing methods. When exposed to specific interactions, for example with water (Tibbits, 2014; Gladman et al., 2016) or UV (Liu et al., 2012), they morph into a programmed shape.

### 3.4. Thin-film manufacturing

When trying to build soft systems at smaller scales, some of the aforementioned manufacturing methods become limited. In this case, techniques adapted from thin-film manufacturing process can be preferred. These techniques, historically developed by the microelectronics industry, allow to create films of precisely controlled thickness that can be cut and stacked in order to produce the required functions. It is critical to create a homogeneous thin layer of material to cast thin-films properly. This is made possible by techniques such as blade coating, where a blade is moved along the manufacturing area, removing excess material to ensure that the thickness of the layer remains constant. Another technique, called spin coating (Hall et al., 2004), consists in spinning a flat bed wetted with the required casting material. After spinning at a sufficient speed, a thin layer of material of constant thickness remains on the bed while the excess material is ejected by centrifugal forces.

Another concept widely used in thin film manufacturing is layer transfer. These techniques allow to build elements on a temporary support and then transfer them on the main film. Roll transfer process (Sharma et al., 2012) allow to attach or detach elements from a layer using the pressure of a rolling cylinder. Pad printing (Krebs, 2009) is a technique also used in the industry for decorating purpose. It uses a transfer pad that collects a pattern formed in a stencil and then transfers it on the destination part or assembly by applying pressure on it.

Using these manufacturing methods with a combination of etching, cutting and lamination steps, it is possible to create planar multi-layered systems that can exhibit out of plane motion (Wood et al., 2008; Jacobsen et al., 2009; Russo et al., 2017).

### 3.5. Shape deposition manufacturing

Shape deposition manufacturing is not actually a manufacturing method, but a concept to create complex and possibly multimaterial structures. It is based on the use of additive and subtractive operations in a sequence to obtain the desired shape. It is not easy to achieve a fully automated process, as multiple manufacturing tools must be available at the same spot. Though, this method allows to make a compromise between the pros and cons of additive and subtractive manufacturing processes. Because there is not a single shape deposition manufacturing method, there is almost no limit in the combination of manufacturing processes, allowing a designer to create structures combining heterogeneous materials (Dollar and Howe, 2006).

### 3.6. Bonding

As exemplified previously, it is difficult to produce complex shapes, with multiple functional geometries and potentially several materials. One of the most straightforward approach to tackle this challenge is to decompose the final system into several parts that are then bound together. While a number of solutions are available in order to bound rigid parts together, this choice is much more limited for soft parts. Indeed, the interface between both parts of a bonded assembly should not exhibit excessive stiffness while still guaranteeing enough adhesion.

A first approach to take advantage of the manufacturing process of the parts themselves. Most soft robotic systems are obtained through the curing of polymeric materials. It is possible to slow the material curing at some point in order to obtain parts in an intermediate state, where they exhibit a solid gel-like behavior, and keep their molded shape. This allows preparing the bonding interface. Then, two separate parts can be merged simply by first adding a thin layer of uncured material similar to a glue, and then finishing the curing process (Ilievski et al., 2011; Marchese et al., 2014). However, this method is limited to chemically compatible materials. Another possibility offered by thermoplastics is mechanical or thermal fusion. These methods are used to create pockets or pleats: several sheets are thermally sealed together to obtain complex closed geometries (Niiyama et al., 2014; Nishioka et al., 2017; Sareen et al., 2017). However, because of the low thermal conductivity of polymers, it is difficult to use this same method on thicker parts. Finally, the last common, possibly most obvious bonding method, is to use glues. The choice of an adapted adhesive for a gluing operation may however be quite challenging. Several criteria affect this choice, including but not limited to chemical compatibility (and adhesion) with both glued material, adhesive strength, flexibility, resistance to solvent and to temperature, setting time, viscosity or contact surface roughness.

Although the aforementioned methods are of an irreversible nature, there are a few reversible bonding processes. This is of very high interest from a robotics standpoint as this reversible bond may be controlled and thus used for some specific applications. This has already been illustrated in the design of climbing (Wang et al., 2013) or reconfigurable (Wang et al., 2014) robots, but could also be adapted to other soft systems.

### 3.7. Architectural considerations

When manufacturing a soft robotic system, material and manufacturing methods are not the only elements that need to be considered. Though most classical “rigid” actuators have the ability to naturally move in both directions about their motion axis, soft actuators are often single action actuators. If the application requires motions in the two directions, the architecture of the system needs to be adapted. If motions in both directions require similar performances, two actuators can be mounted in an antagonistic fashion, as in bio-mechanical systems (Verrelst et al., 2005). An advantage of this configuration is that when the combined actuators exhibit non-linear stiffness, both the position of the antagonistic mechanism and its global stiffness can be controlled separately (Vanderborght et al., 2013). When considering a system with multiple degrees of freedom, more than two actuators can be mounted antagonistically in order to create complex motion (Bishop-Moser et al., 2012; Martinez et al., 2013). Alternatively, some actuators may use their internal stiffness or external elastic elements to return back to their initial configuration. While this solution is used quite often for soft fluidic actuators (e.g., Mosadegh et al., 2014; Calderón et al., 2016) or for shape memory material-actuated systems (e.g., Hines et al., 2012; Jin et al., 2016), the control of the return motion is more limited.

Another major issue for some soft actuator designs is buckling. Although this phenomenon also exists in stiff structures, the low stiffness of some soft actuators render them prone to this elastic instability. For this reason, it is preferable to use actuators with high length to bending modulus ratio in pulling motion than in pushing motion, as buckling is generated by compression forces. Finally, some actuators rely on internal stress, as for instance coiled artificial muscles (Haines et al., 2014) or some SMA-based systems (Meisel et al., 2015). From a design standpoint, such systems have to be prestressed at manufacturing time, and later be able to conserve this prestress in order to avoid the destruction of either the actuator or the rest of the structure.

Finally, some systems use the flexibility offered by tendon-like elements (Manti et al., 2015; Rateni et al., 2015; Mutlu et al., 2016) in order to transmit forces between the actuator and the structure. This architecture allows the mechanical elements to accommodate unstructured environments while being actuated by single degree of freedom actuators such as classical motors or synthetic muscles.

## 4. Conclusions and perspectives

### 4.1. Various novel tools with pros and cons

Soft robotics is a relatively recent topic. However, a wide range of systems have already been developed, with a great number of applications (Laschi et al., 2016). This technological push has been closely tied to the development of new manufacturing techniques, allowing to produce increasingly performant devices, that can even include additional functions in top of their purely soft-mechanical behavior. The introduction of new innovative materials, such as self-healing polymers (Terryn et al., 2015, 2018), or biocompatible elastomers will also lead to further expansions for soft robotics the field of medical applications. The underlying manufacturing technologies already allow to tackle some of the challenges emerging from the use of soft materials in robotics, and they will go on developing with the advances of several scientific communities converging on soft robotics.

A wide range of manufacturing methods have been proposed throughout the literature, ranging from methods adapted from their industrial couterparts to new emerging solutions that could help shaping new designs in the future. Table 1 sums up the main manufacturing methods presented in this paper with their principal features and applications. Because of the wide diversity of the produced parts in terms of size, geometry and material properties, manufacturing methods cannot be readlily compared.

**Table 1:** Summary of the principal manufacturing methods presented in this article, the typical scale range of the parts produced and the main applications and features of each method.

**Manufacturing method**	**Part scale [mm]**	**Applications**	**Advantages**	**Drawbacks**	
Gravity molding	10^3^	Structural elements Fluidic actuators	Low implementation cost Wide adaptability	Degassing required Long molding time
Centrifugal molding	10^-3^-10^1^	Microfluidics, thin systems	High precision details Degassing without vacuum	More adapted to thin parts Internal volumes difficult
Vacuum molding	10^-3^-10^1^	Microfluidics Systems with small details	High precision details	Complex positioning of bubble traps for some details
Spin casting	1-10^2^	Void elements with thin walls	Void chamber easy to cast Simple molds	Internal walls with no details Wall thickness control
Dip coating	1-10^2^	Void elements with thin walls Balloons, gloves, bellows	Void chamber easy to cast Simple molds	External walls with no details Streaks/excessive thickness
Vacuum infusion	10^1^-10^4^	Fiber reinforced elements	Low implementation cost Very large parts	Details only on one side Relies on reinforcement	
Stereolithography	10^-1^-10^2^	Prototyping, do-it-yourself Low-strength parts manufacturing	Low cost Large range of materials	Poor mechanical properties High surface roughness Long manufacturing time
Inkjet printing	10^-2^-10^2^	Prototyping, 4D printing, Small series manufacturing	Multi-material patterning Low production time	Highly relying on scaffoldings High material cost
Direct ink writing	10^-3^-1	Micro-machining, 4D printing, Precision manufacturing	High precision Scaffoldings optional	Long production time
Continuous liquid interface production	10^-2^-10^1^	Prototyping, Small series manufacturing	Low production time Low surface roughness	Mono-material
Spin coating	10^-4^-10-^1^	Thin film	Fast and simple	Thickness control only Flat film
Tape coating	10^-3^-1	Thin film	Liquid or powder Flat or cylindrical film	Thickness control only
Pad printing	10^-3^-10^-1^	Thin film, Decoration	2D patterns Works on irregular surfaces	Stencil required
Stereolithography	10^-2^-10^3^	Prototyping Small series manufacturing	Low surface roughness Large range of materials	Mono-material
Fused deposition modeling	10^-1^-10^2^	Prototyping, do-it-yourself Low-strength parts manufacturing	Low cost Large range of materials	Poor mechanical properties High surface roughness Long manufacturing time
Inkjet printing	10^-2^-10^2^	Prototyping, 4D printing, Small series manufacturing	Multi-material patterning Low production time	Highly relying on scaffoldings High material cost
Direct ink writing	10^-3^-1	Micro-machining, 4D printing, Precision manufacturing	High precision Scaffoldings optional	Long production time
Continuous liquid interface production	10^-2^-10^1^	Prototyping, Small series manufacturing	Low production time Low surface roughness	Mono-material
Spin coating	10^-4^-10^-1^	Thin film	Fast and simple	Thickness control only Flat film
Tape coating	10^-3^-1	Thin film	Liquid or powder Flat or cylindrical film	Thickness control only
Pad printing	10^-3^-10^-1^	Thin film, Decoration	2D patterns Works on irregular surfaces	Stencil required

In the first block of the table are gathered the principal molding techniques described in section 3.1. These methods give the designer the best control over material properties but requires the constuction of molds. The second block presents AM methods detailed in section 3.3. They offer broad ranges of possibilities to create freeform geometries that could not be obtained otherwise. However the choice in material types is generally more restricted than with molding techniques. The last block shows more specific techniques for manufacturing multilayer thin-film systems as discussed in section 3.4.

### 4.2. Modeling and design perspectives

Modeling is of central importance for characterizing existing soft devices, but also for the design and the synthesis of novel soft robots. Soft robots can be best modeled as continuous deformable media, but analytic models are difficult (if possible) to derive for soft robots exhibiting geometric and behavorial non-linearities. Advances in the field of compliant mechanisms have provided possible approaches for modeling the load-displacement behavior of devices comprising flexure members. The pseudo-rigid body model approach (Howell, 2001) consists in setting a correspondence between deformable members and an equivalent rigid-body mechanism where flexures are modeled as massless spring elements. Other models can be chosen depending on the deflection level, such as Sen and Awtar (2013) for small to medium deflections, and Holst et al. (2011) for larger displacements, but are however limited to 1D beam shape geometries. Most research toward analytic modeling of soft robots is oriented toward fiber-reinforced structures, such as bending actuators (Memarian et al., 2015a; Wang et al., 2017) or fiber-reinforced elastofluidic enclosures (Krishnan et al., 2015; Bruder et al., 2017).

**Figure 10:**
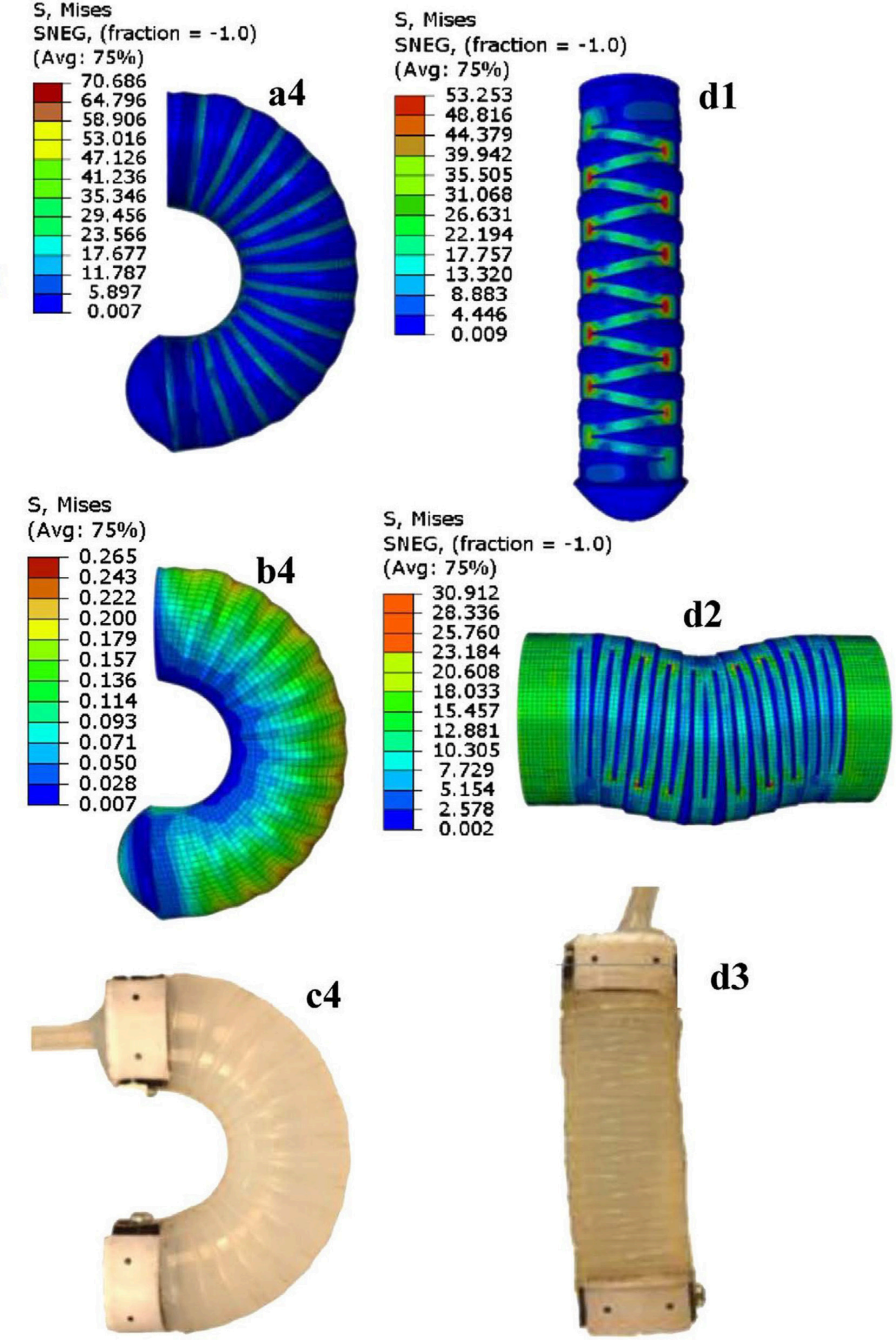
Example of a soft tubular actuators with external reinforcements, as presented in Agarwal et al. (2016). The left column shows a bending variant of the actuator while the right column shows a linear extension one. On the top, FEM simulation results are presented: (a4) and (b4) represent the von Mises stress, respectively for the full actuator and for the soft core; (d1) and (d2) show the von Mises stress for the linear actuator, respectively in free and blocked extension. The pictures at the bottom show the corresponding experimental results. Image reproduced from Springer Nature with permission from J. Paik.

In most case, however, an analytical model is not available. Then, iterative finite element methods (FEM) can be used to solve the equation of continuum mechanics. Although computationally expensive, they only offer little parametric design insight (Sen and Awtar, 2013) and are more adapted at the design stage to check the validity of a closed-form model, or to provide comparative results with experiments (as shown in Figure 10). Recently, however, real-time FEM for soft robot simulation and control has been proposed (Duriez, 2013). The computational speed of such approach comes however with some associated tradeoffs such as simplistic material constitutive laws and low dynamics. Other approaches adapted from physics simulation and computer graphics such as those proposed by Hiller and Lipson (2014) allow the modeling of voxelized structures built with heterogeneous material properties.

The synthesis of new soft robots can also be adapted from classical methods already available in the field of mechanism. Hopkins et al. (2015) presents a synthesis and analysis methods for generating soft parallel robots using pneumatic trichamber actuators. Other methods relying on topological optimization can also be useful in the design of soft robots. First adapted from structural cases (Bendsøe and Kikuchi, 1988) to compliant mechanisms synthesis (Yin and Ananthasuresh, 2003), they can be used to synthesize soft and multimaterial mechanisms (Hiller and Lipson, 2012; Meisel et al., 2013). Finally, bioinspiration is a vast source of inspiration for soft robots (Kim et al., 2013) as it has fueled the field since its early beginnings.

### 4.3. Instrumentation and control perspectives

Designers of soft robots also face many other challenges that are yet to be properly addressed (Rus and Tolley, 2015; Laschi et al., 2016). Some ambitious challenges lie in the instrumentation and control of such systems, and also in the design of embedded components adapted to the specificities of soft robotics. Multimaterial manufacturing makes it today possible to embed various elements in the soft matrix of the robot, in order to improve the integration of the functionalities. Modularity increases Onal and Rus (2012), with soft robots including their own pressure sources (Onal et al., 2011) and control valves (Marchese et al., 2011). The inclusion of channels for liquid metal injection (Park et al., 2012; Farrow and Correll, 2015) or the embedding of conductive hydrogels and electroactive fillers in flexible materials (Larson et al., 2016), open perspectives in soft electronics (Correll et al., 2014), a step further in the development of sensing systems for soft robots. To date, reliable sensors able to measure the state of a soft system only provide partial information on the state of the system, though more and more solutions have been proposed, using for example liquid metals (Park et al., 2012; White et al., 2017) or microstructured metal on polymeric substrate (Araromi et al., 2016; Atalay et al., 2017). The control of soft robots is also a very open challenge, and it has to be acknowledged that modeling soft device for realtime control is currently at a very early stage in spite of noticeable contributions (Duriez, 2013; Coevoet et al., 2017).

All these challenges may currently limit the adoption of soft robots technologies in the industry, at least in the short term. However, they are currently an extraordinary source of investigations for research groups, suggesting many improvements in the years to come.

## Author contributions

FS has made the in-depth bibliographic research and has written the article body. OP and LB have reviewed the introduction and the sections 2, 3, and 4. BB has written the introduction, and reviewed the whole article.

### Conflict of interest statement

The authors declare that the research was conducted in the absence of any commercial or financial relationships that could be construed as a potential conflict of interest.
